# Alginate Oligosaccharides Enhance Antioxidant Status and Intestinal Health by Modulating the Gut Microbiota in Weaned Piglets

**DOI:** 10.3390/ijms25158029

**Published:** 2024-07-23

**Authors:** Ming Liu, Xiong Deng, Yong Zhao, Nadia Everaert, Hongfu Zhang, Bing Xia, Martine Schroyen

**Affiliations:** 1 Animal Science and Technology College, Beijing University of Agriculture, Beijing 102206, China; liuming@bua.edu.cn (M.L.);; 2 Precision Livestock and Nutrition Unit, Gembloux Agro-Bio Tech, University of Liège, Passage des Déportés 2, 5030 Gembloux, Belgium; 3 State Key Laboratory of Animal Nutrition, Institute of Animal Science, Chinese Academy of Agricultural Sciences, Beijing 100193, Chinazhanghongfu@caas.cn (H.Z.); 4 Nutrition and Animal Microbiota Ecosystems Laboratory, Department of Biosystems, KU Leuven, 3001 Leuven, Belgium

**Keywords:** alginate oligosaccharides, weaned piglets, intestinal barrier function, antioxidant capacity, gut microbiota

## Abstract

Alginate oligosaccharides (AOSs), which are an attractive feed additive for animal production, exhibit pleiotropic bioactivities. In the present study, we investigated graded doses of AOS-mediated alterations in the physiological responses of piglets by determining the intestinal architecture, barrier function, and microbiota. A total of 144 weaned piglets were allocated into four dietary treatments in a completely random design, which included a control diet (CON) and three treated diets formulated with 250 mg/kg (AOS250), 500 mg/kg (AOS500), and 1000 mg/kg AOS (AOS1000), respectively. The trial was carried out for 28 days. Our results showed that AOS treatment reinforced the intestinal barrier function by increasing the ileal villus height, density, and fold, as well as the expression of tight junction proteins, especially at the dose of 500 mg/kg AOS. Meanwhile, supplementations with AOSs showed positive effects on enhancing antioxidant capacity and alleviating intestinal inflammation by elevating the levels of antioxidant enzymes and inhibiting excessive inflammatory cytokines. The DESeq2 analysis showed that AOS supplementation inhibited the growth of harmful bacteria *Helicobacter* and *Escherichia_Shigella* and enhanced the relative abundance of *Faecalibacterium* and *Veillonella*. Collectively, these findings suggested that AOSs have beneficial effects on growth performance, antioxidant capacity, and gut health in piglets.

## 1. Introduction

In the modern swine industry, piglets are often confronted with multiple challenges. Early weaning stress is one of the most significant challenges for piglets, as weaning deprives the passive immune protection conferred by sow milk and causes intestinal mucosal barrier dysfunctions that lead to reduced growth, diarrhea, and increased mortality [1]. Although the use of antibiotics can effectively alleviate the problems induced by weaning stress, the abuse of antibiotics leads to animal intestinal microbiota dysbiosis, drug resistance, and the risk of antibiotic residues in animal products [2,3,4]. Therefore, it is urgent to develop antibiotic alternatives to maintain pig health during the postweaning period.

Alginate is an acidic polysaccharide consisting of β-D-mannuronic acid and α-L-guluronic acid linked via 1,4-glycosidic bonds [5]. Although alginate supports a range of health-promoting activities, such as immune-enhancing and antioxidant activities [6], the multifaceted development and application of the polymer have been greatly hampered by its high molecular weight, high viscosity, and low solubility [7]. Alginate oligosaccharides (AOSs) are the degradation products of alginate extracted from brown algae with a higher water solubility and lower solution viscosity compared with alginate [8]. AOSs exhibit pleiotropic bioactivities, including antioxidant, anti-inflammatory, and anti-bacterial effects [9,10]. AOSs could block H_2_O_2_-induced oxidative stress [11] and alleviate the disrupted endoplasmic reticulum (ER) and mitochondria of cells, damaged cell membranes and microvilli, and promote the recovery of intestinal functions [12]. AOSs attenuated the inflammatory responses in a dextran sulfate sodium-induced colitis mice model by inhibiting the increasing expression of inflammatory markers and modulating gut microbiota [13,14]. Moreover, it has been demonstrated that AOS supplementation could regulate the gut microbiota via increasing the relative abundance of beneficial bacteria while inhibiting harmful bacteria, and therefore maintain intestinal epithelial integrity [15].

In a weaned pig model, studies have shown that AOSs protect the intestinal mucosa from ETEC (Enterotoxigenic *Escherichia coli*)-induced epithelial and inflammatory injury by reducing both mitochondria-dependent and tumor necrosis factor receptor 1-dependent apoptosis [16] and preventing the activation of NF-κB [17]. Given the reported ability of AOSs to improve intestinal barrier function, it may be an attractive feed additive for use in swine production. However, there has been scarce knowledge about the effects of AOSs on growth performance, serum parameters, and gut health. The potential optimal dose of AOSs used in weaned piglets is also unknown. Accordingly, the present study set out to investigate the effects of graded doses of AOS supplementation on the growth performance, redox status, inflammatory response, intestinal barrier function, and gut microbiota of piglets, and to explore the role of gut microbiota in the physiological alterations induced by AOSs.

## 2. Results

### 2.1. Supplementations with 500 mg/kg AOS Elevated Body Weight

There was no difference in initial body weight (BW) among the treatments (Table 1). The BW on day 14 was comparable between the AOS500 and CON groups. However, the AOS500 group showed a significant increase in BW on day 28. Piglets in the AOS1000 group had a higher BW on day 28, but the difference was insignificant (Table 1). Compared with the CON group, dietary supplementation with AOS500 increased the average daily gain (ADG) during days 15 to 28 and days 1 to 28. AOS1000 supplementation tended to increase ADG compared with the CON group during day 1 to 14 and day 1 to 28 (Table 1). No significant effects of AOSs on average daily feed intake (ADFI) and the gain to feed ratio (G:F) were observed throughout the trial (Table 1).

### 2.2. AOSs Enhanced the Intestinal Barrier Function

**Figure 1:**
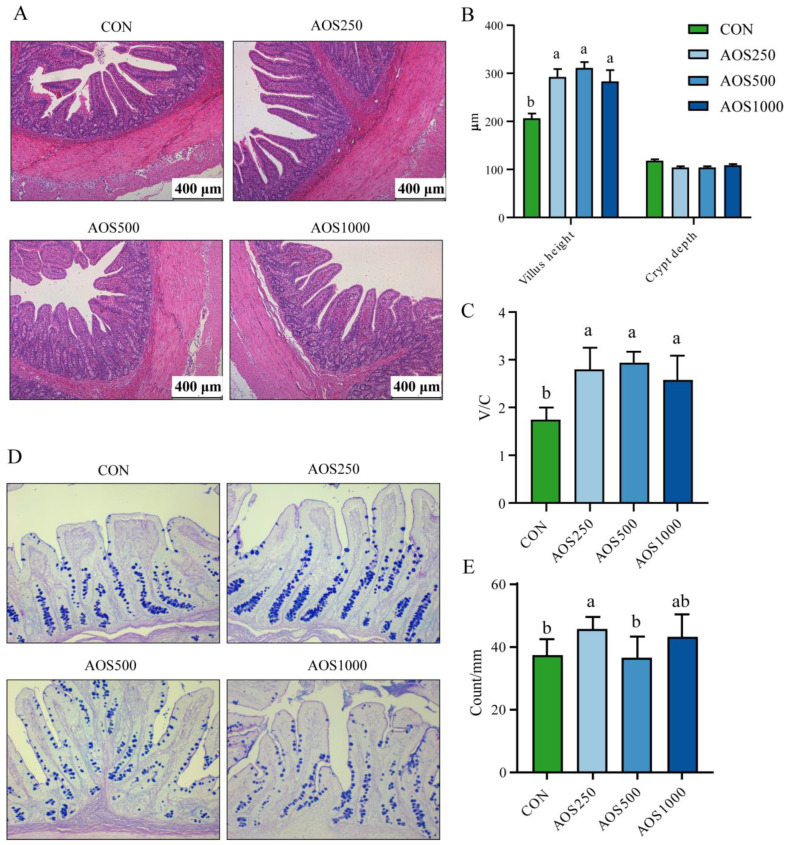
Effects of AOS on ileal morphology. (**A**) Representative images of HE staining in the ileum of piglets. (**B**) The villus height, crypt depth, and (**C**) V/C of the ileum in the piglets. (**D**) Representative images of AB-PAS staining in the ileum of piglets. The scale bar is 400 μm. (**E**) The counts of goblet cells in the ileum of the piglets. AOS: alginate oligosaccharide, V/C: villus height to crypt depth ratio. Data are presented as means ± SEM. Different letters between the groups are statistically significant (*p* < 0.05).

We assessed the ileal morphology of piglets through HE staining (Figure 1A). The AOS treatments significantly elevated the ileal villus height and villus height/crypt depth (V/C) compared with the CON group (Figure 1B,C). However, the ileal crypt depth did not markedly differ among the four distinct groups (Figure 1B). To investigate the effect of AOSs on mucus secretion, AB-PAS staining on ileal mucosa was performed (Figure 1D). The AOS supplementation groups increased the number of goblet cells. Supplementations of 250 mg/kg AOS significantly increased ileal goblet cells. The number of goblet cells in the AOS1000 group showed an upward trend, but this difference did not reach statistical significance (Figure 1E).

**Figure 2:**
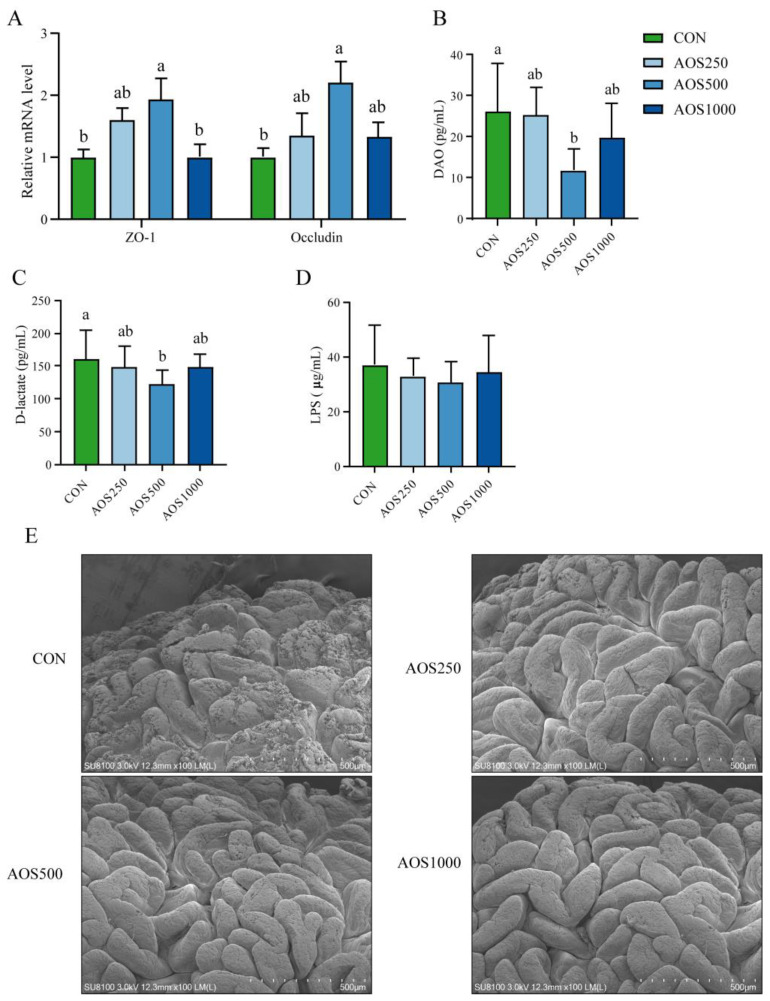
Effects of AOSs on the ileal barrier function. (**A**) Relative mRNA expression of ZO-1 and Occludin in the ileum. The concentration of (**B**) DAO, (**C**) D-lactate, and (**D**) LPS in serum. (**E**) Scanning electron microscopy analysis of ileal mucosa epithelium in piglets. DAO: diamine oxidase. Data are presented as means ± SE. Different letters between the groups are statistically significant (*p* < 0.05).

AOS500 supplementation substantially elevated the expression of *ZO-1* and *Occludin*, whereas AOS250 supplementation tended to increase the gene expression of the tight junction proteins (Figure 2A). The AOS500 group exhibited a significant reduction in diamine oxidase (DAO) and D-lactate levels compared with the CON group. The AOS250 and AOS1000 groups displayed a decreasing trend in the levels of DAO and D-lactate (Figure 2B,C). There were, however, no changes in the concentration of LPS in serum among the four groups (Figure 2D).

To evaluate the effect of AOSs on the ileal epithelium and microvilli of piglets, a scanning electron microscope (SEM) was utilized for observing the ileum. It was shown that weaning stress induces significant damage to the ileal epithelium and reduced the microvilli density (Figure 2E). Supplementation with AOSs induced an increase in villi density and villi fold. In the AOS treatment groups, the lesions showed a tidier microvilli morphology and more intact ileal epithelium.

### 2.3. AOS Regulated Redox Status and Systemic Immunity

**Figure 3:**
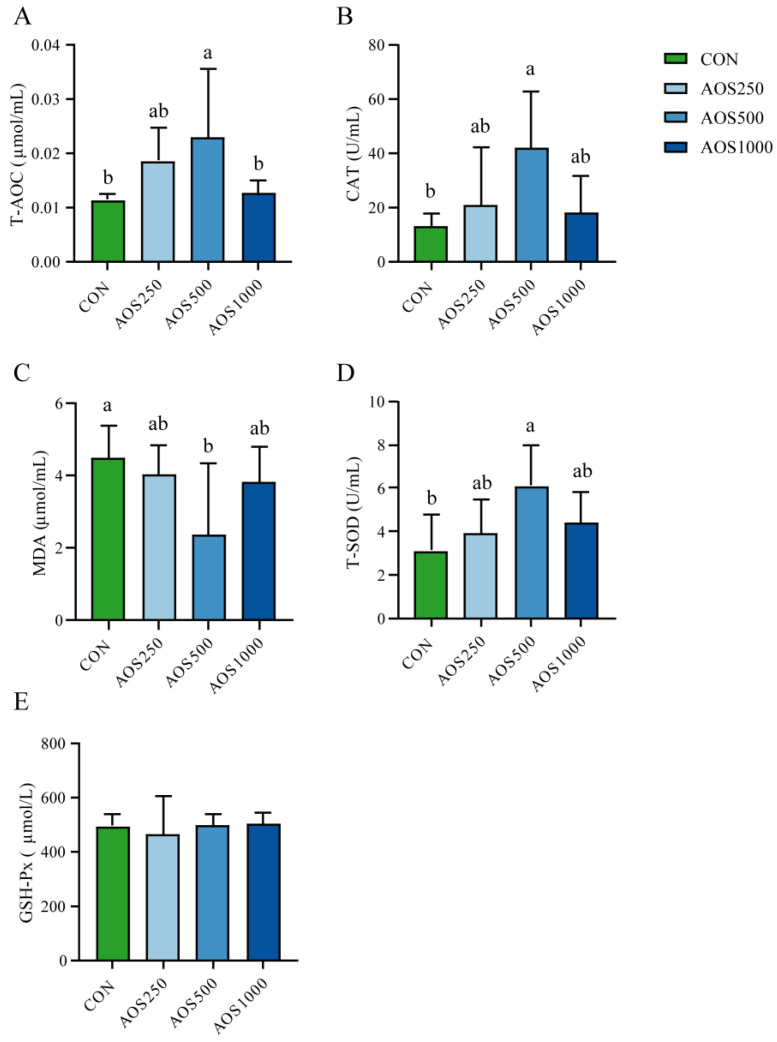
Effects of AOS on the antioxidant capacity. The concentrations of (**A**) T-AOC, (**B**) CAT, (**C**) MDA, (**D**) T-SOD, and (**E**) GSH-Px in serum. T-AOC: total antioxidant capacity, CAT: catalase, MDA: malondialdehyde, T-SOD: total superoxide dismutase, GSH-Px: glutathione peroxidase. Data are presented as means ± SE. Different letters between the groups are statistically significant (*p* < 0.05).

In order to evaluate the redox status of weaned piglets fed with AOSs, we detected serum total antioxidant capacity (T-AOC), antioxidant enzymes including catalase (CAT), total superoxide dismutase (T-SOD), and glutathione peroxidase (GSH-Px). Excessive ROS leads to increased peroxidation, resulting in elevated levels of MDA in the serum. As shown in Figure 3A–D, compared with the CON group, the activities of T-AOC, CAT, and T-SOD were elevated, whereas the malondialdehyde (MDA) level was reduced in the AOS500 group. Supplementation with AOS250 tended to enhance the activities of T-AOC, CAT, and T-SOD compared with the CON group (Figure 3A). AOS250 and AOS1000 had a tendency to reduce the MDA level in the serum relative to the CON group (Figure 3C). The activity of GSH-Px in the AOS-treated groups was not significantly altered when compared to the CON group (Figure 3E).

**Figure 4:**
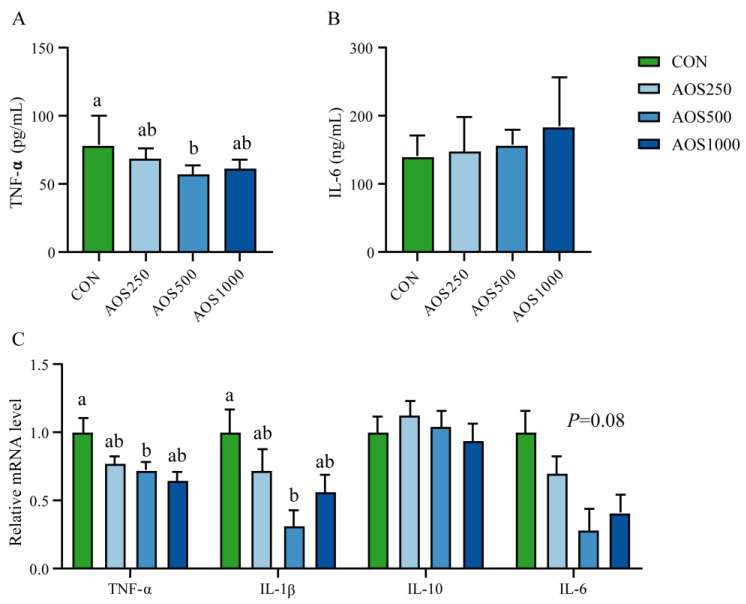
Effects of AOS on immune response of piglets. The levels of (**A**) TNF-α and (**B**) IL-6 in serum. (**C**) Relative mRNA expression of TNF-α, IL-1ꞵ, IL-10, and IL-6 in the ileum. Data are presented as means ± SE. Different letters between the groups are statistically significant (*p* < 0.05).

Cytokines including interleukin-1ꞵ (IL-1β), interleukin-6 (IL-6), interleukin-10 (IL-10), and tumor necrosis factor-α (TNF-α) were measured to assess the inflammatory status in piglets. As shown in Figure 4A,C, AOS500 supplementation significantly reduced the levels of TNF-α in the serum and ileum as compared with the CON group. Dietary supplementation with AOS500 also reduced the expression of IL-6 in the ileum, but the difference was insignificant. Additionally, dietary AOS500 markedly decreased the expression of IL-1β, whereas AOS250 and AOS1000 tended to reduce the mRNA level of IL-1β (Figure 4C). Furthermore, AOS250 and AOS1000 supplementation had a tendency to decrease the expression of TNF-α (Figure 4C).

### 2.4. AOS Feeding Altered Microbial Composition of the Colonic Luminal Contents

**Figure 5:**
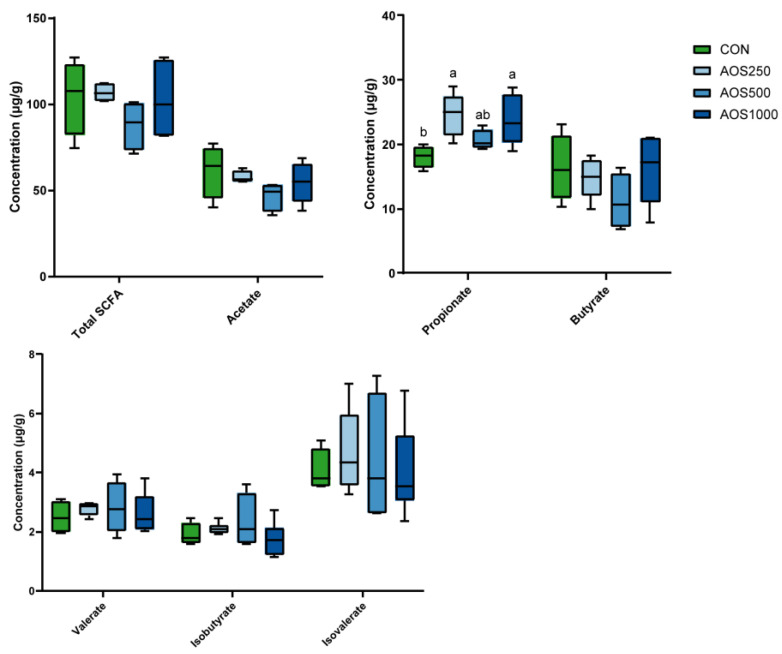
The levels of SCFAs in the colonic luminal contents. Data are presented as min to max. Different letters between the groups are statistically significant (*p* < 0.05).

The concentrations of colonic short chain fatty acids (SCFAs) were determined. As illustrated in Figure 5, both AOS500 and AOS1000 groups had significantly higher levels of propionate in the colon. Piglets in the three AOS treatment and control groups had a homogeneous production of total SCFAs, acetate, butyrate, isobutyrate, valerate, and isovalerate.

In this study, the 16S rDNA gene-sequencing analysis was conducted to investigate the effect of AOSs on the gut microbial diversity in piglets. Piglets in the CON and AOS supplementation groups had no significant difference in the α-diversity, including the Chao1 index, Shannon index, and Simpson index, except for the ACE index. The ACE index of the AOS1000 group was lower than that of the CON group (Figure S1A–D). PCoA based on Bray–Curtis distance matrices showed that piglets had a homogeneous community composition among the four groups (Figure S1E). The Partial Least Squares Discriminant Analysis (PLS-DA) model exhibited that microbial structures in the AOS500 and AOS1000 groups displayed a different dispersion from the CON group (Figure S1F).

**Figure 6:**
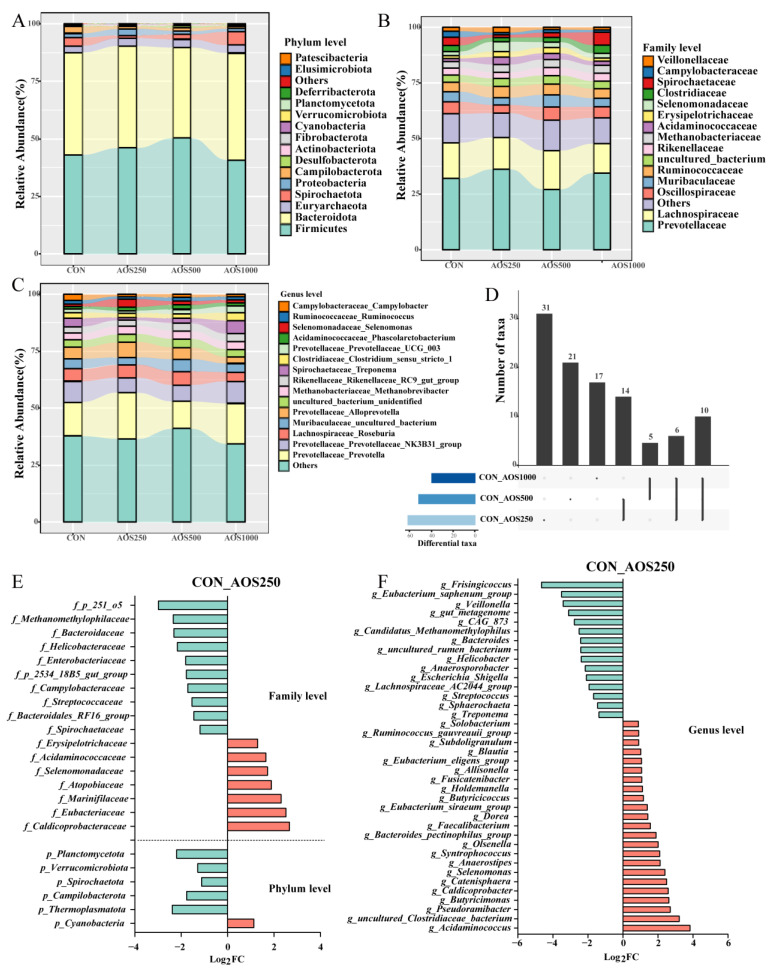
The overall composition of microbiota in the colon of piglets. The relative abundance of microbial composition at the phylum (**A**), family (**B**), and genus (**C**) level. The overlapped taxa among the three AOS groups (**D**). Differences in microbial community between the CON group and the AOS250 group at the phylum and family (**E**) and genus (**F**) level.

**Figure 7:**
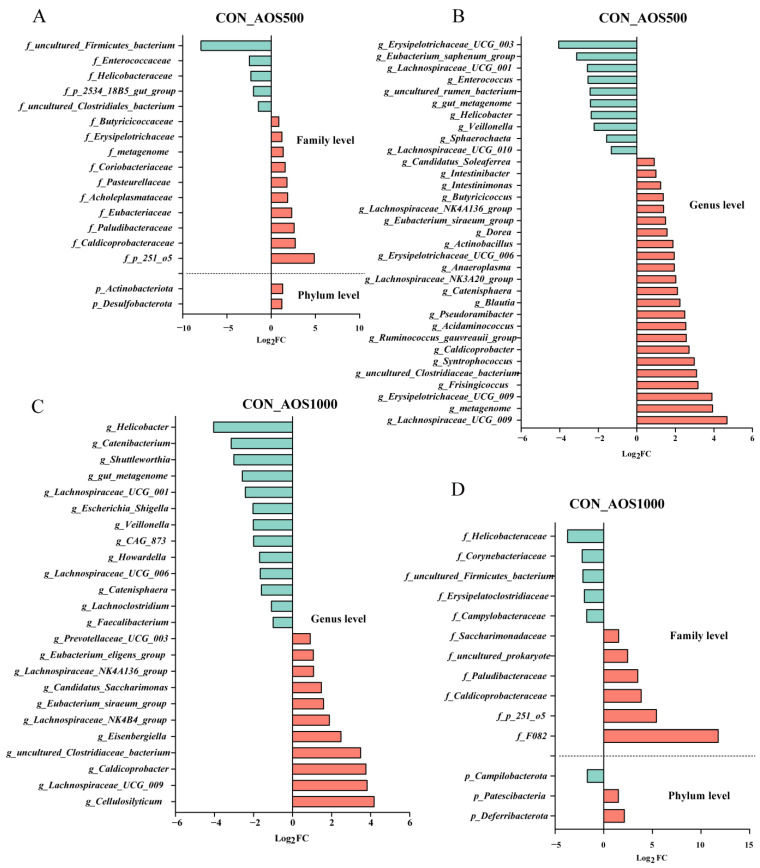
Effects of AOS supplementation on microbiota. Differences in colonic microbiota between the CON group and the AOS500 group at the phylum and family (**A**) and genus (**B**) level. Differences in colonic microbiota between the CON group and the AOS1000 group at the phylum and family (**C**) and genus (**D**) level.

At the phylum level, *Firmicutes*, *Bacteroidetes*, and *Euryarchaeota* predominantly constituted the colonic microbiota of the piglets (Figure 6A). In Figure 7B,C, the structure of the top 15 abundant taxa within the groups at the levels of family and genera is shown. We found the abundance of *Prevotellaceae*, *Lachnospiraceae*, and *Oscillospiraceae* as the dominating family in the colonic microbiota of the piglets. The genera *Prevotella*, *Prevotellaceae_NK3B31_group*, and *Alloprevotella* from the family *Prevotellaceae* and *Roseburia* from the family *Lachnospiraceae* were dominant in the colon of piglets.

Differentially abundant taxa were analyzed using the DESeq2 algorithm. The overlapped taxa sets were analyzed using the “UpSetR” package among the three AOS groups (Figure 6D). A total of 10 commonly altered bacterial taxa (*f_Caldicoprobacteraceae, f_Helicobacteraceae, f_p_251_o5, g_uncultured_Clostridiaceae_bacterium*, *g_Caldicoprobacter*, *g_Catenisphaera*, *g_Eubacterium_siraeum_group*, *g_Helicobacter*, *g_gut_metagenome*, *g_Veillonella*) of graded levels of AOSs were screened out. We identified five (*f_Paludibacteraceae, f_uncultured_Firmicutes_bacterium*, *g_Lachnospiraceae_UCG_009*, *g_Lachnospiraceae_NK4A136_group*, and *g_Lachnospiraceae_UCG_001*) and six (*p_Campilobacterota, f_Campylobacteraceae*, *g_Faecalibacterium*, *g_Eubacterium_eligens_group*, *g_Escherichia_Shigella*, and *g_CAG_873*) overlaps of differentially abundant bacteria between the AOS1000 versus AOS500 treatment groups and the AOS1000 versus AOS250 treatment groups, respectively, as shown in Figure 6D. There were 14 overlaps of significant changes (*f_Eubacteriaceae, f_Erysipelotrichaceae, f_p_2534_18B5_gut_group*, *g_Acidaminococcus*, *g_Pseudoramibacter*, *g_Syntrophococcus*, *g_Dorea*, *g_Butyricicoccus*, *g_Blautia*, *g_Ruminococcus_gauvreauii_group*, *g_Sphaerochaeta*, *g_uncultured_rumen_bacterium*, *g_Eubacterium_saphenum_group*, and *g_Frisingicoccus*) between the AOS500 and AOS250 treated piglets.

The AOS250 group showed a significant increase in the abundance of *Anaerostipes* and *Butyricimonas* compared to the CON group, whereas *Streptococcus* was less abundant (Figure 6E). In Figure 7A, significant increases in abundance were noted for *Actinobacteriota* and *Desulfobacterota* at the phylum level in the AOS500 group compared with the CON group. At the genus level, the AOS500 group had enriched the relative abundance of *Intestinibacter*, whereas the relative abundance of *Lachnospiraceae_UCG_010* and *Lachnospiraceae_NK3A20_group* was dampened (Figure 7B). In Figure 7D, it can be seen that the AOS1000 group had a higher proportion of *Patescibacteria* and *Deferribacterota* compared with the CON group. AOS1000 supplementation reduced the abundance of *Howardella* and *Shuttleworthia*.

### 2.5. Correlation Analysis between Intestinal Microbiota and Barrier Related Indicators

**Figure 8:**
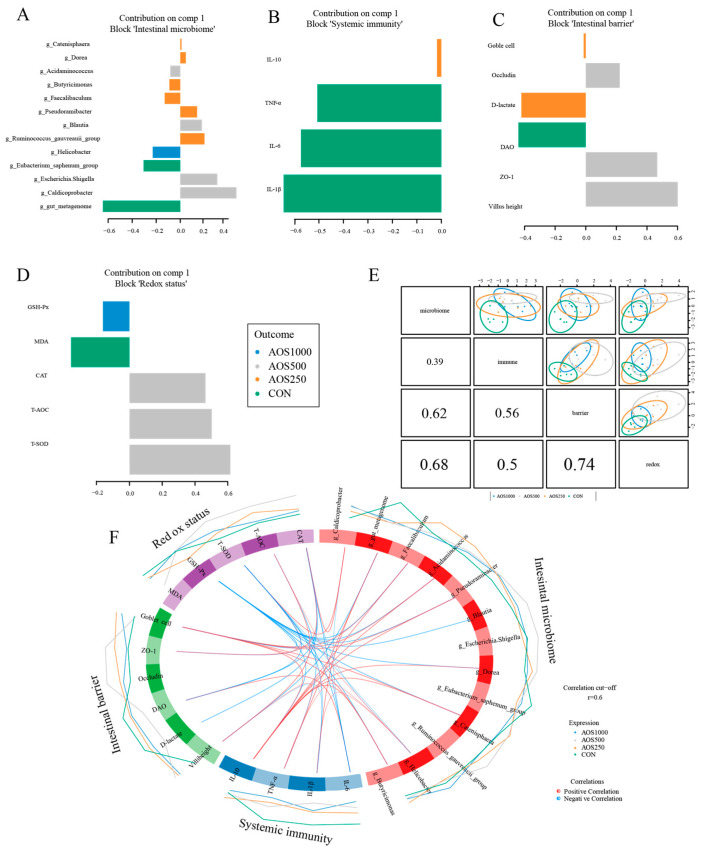
Correlation analysis for intestinal barrier, redox status, systemic immunity, and intestinal microbiome. The top loading weights of each of the selected variables on component 1 (**A**–**D**). A global overview of the correlation structure at the four components (**E**). The circus plot displaying correlations among the four components (**F**).

Integration using the DIABLO revealed the the correlation among intestinal barrier function, antioxidant status, immune response, and microbial groups. The selected variables of four components were visualized in loading plot (Fig. 8A-D). The top 5 predictors in the microbiome component were gut_metagenome, Caldicoprobacter, Escherichia_Shigella, Eubacterium_saphenum_group, and Helicobacter. In the intestinal barrier, the selected key predictors (top 3) were villus height, *ZO-1*, and DAO. In the antioxidant status component, the top 3 loading weights of the selected variable were T-SOD, T-AOC, and CAT. We found a strong correlation among the block microbiome, intestinal barrier, and antioxidant status using DIABLO analysis (Figure 8E). 

Our results revealed that five genera (Blautia, Pseudoramibacter, Acidaminococcus, Faecalibaculum, and Butyricimonas) had a negative correlation with GSH-Px. The abundance of Caldicorprobacter was positively correlated with the antioxidant enzymes CAT and T-SOD. Furthermore, T-SOD showed a negative correlation with IL-1β and IL-6, whereas showed a positive correlation with *ZO-1*. Consistently, we found that CAT was positively correlated with *ZO-1*, but negatively correlated with intestinal permeability indices DAO and D-lactate as well as cytokine IL-6. We found that T-AOC was positively correlated with villus height, whereas negatively correlated with TNF-α. It identified that IL-10 was positively correlated with four genera (Pseudoramibacter, Acidaminococcus, Faecalibaculum, and Butyricimonas) (Figure 8F).

## 3. Discussion

Given that antibiotics have detrimental impacts on food safety and human health, alternatives to antibiotics are urgently needed in animal agriculture [18]. Prebiotics, especially oligosaccharides, exhibit various beneficial effects on the host [19,20]. The aim of this experiment was to examine the impact of AOSs on growth performance, antioxidant activity, inflammatory response, and intestinal mucosal barrier function. In the current study, diets with different AOS levels increased the ADFI for weaned piglets from d 1–28, suggesting that the dietary inclusion of AOSs may promote appetites in weaned piglets. However, additional studies are needed to confirm this hypothesis. Furthermore, AOSs were more effective at the intermediate inclusion (500 mg/kg) in eliciting a positive response, which is reflected in the elevated ADG and final BW throughout the trial. Given dietary AOS500 supplementation in the diet increased the ADG and final BW, we suppose that a 500 mg/kg level of AOSs in the diet may be an optimal dose for weaned piglets. However, a study from Wan et al. [21] has shown that supplementation with 100 mg/kg or 200 mg/kg AOS for 2 weeks improved the ADG. This could be due to differences in the source of AOSs or the duration of the trial between these studies. Finding the optimal dose requires further research using a wider grade of doses of AOSs (from 100 mg/kg to 1000 mg/kg) to be confirmed. Previous research also demonstrated that functional oligosaccharides, including sialyllactose, xylooligosaccharides, and dietary fiber, have positive dose effects on growth performance in piglets [22,23] and broilers [24].

The intestinal epithelium facilitates the digestion and absorption of nutrients and forms a barrier to protect the internal environment of the intestines against invasions by pathogenic microorganisms and exogenous toxin attacks [25]. The small intestine is the primary location for nutrient uptake, where it identifies the components of the intestinal chyme to transmit essential nutrients throughout the body. Intestinal morphology is a critical predictor of health status, in which villus height and the ratio of villus height to crypt depth play an essential role in nutrient digestion and absorption as well as cell maturation rate. It is therefore vital for the growth and development of postweaning piglets to maintain a normal intestinal architecture. Growth falters in weaned piglets partly due to a compromised intestinal architecture, such as villus atrophy and crypt hyperplasia [26,27,28]. Zha et al. [29] demonstrated that dietary mannan oligosaccharide selenium supplementation significantly reduced intestinal barrier permeability and improved barrier function. Wang et al. [30] found that *Bacillus amyloliquefaciens* supplementation is effective in preventing intestinal epithelial damage in LPS-challenged piglets. Such an improved ileal morphology was observed in pigs supplemented with functional oligosaccharides [23,31] and probiotics [32]. Our study demonstrated that AOS consumption improved the ileal architecture, including an increased villus height and V/C ratio, which may account for the mild increased growth performance. These findings are consistent with those of Wan [33], who found that the dietary inclusion of AOSs could increase the duodenal and jejunal V/C ratio, as well as the villus surface area in the jejunum.

The intestinal mucosal barrier serves as a robust physical demarcation separating the host from pernicious microbes, controlling intestinal inflammation and homeostasis. The intestinal barrier is compromised in conditions such as weaning, inflammation, and various diseases. Tight junction proteins (TJs), as the crucial components of the intestinal epithelial barrier, maintain the integrity and selective permeability of the intestinal epithelium [34]. TJs are composed of transmembrane proteins, including TJ-associated marvel proteins such as Occludin, Claudins, and ZO proteins as well as junctional adhesion molecules [34]. As shown by our results, AOSs at a level of 500 mg/kg in the diet showed a positive impact on improving the ileal epithelial barrier by elevating the expression of *Occludin* and *ZO-1*. The function of the intestinal epithelial barrier is also connected to various factors, such as D-lactate, endotoxins, and DAO in serum [35]. DAO is an intracellular enzyme synthesized by intestinal epithelial cells [36] and D-lactate is a metabolite of intestinal bacteria [37]. Both of them will be circulated in the blood upon the disruption of the intestinal epithelial barrier. In our study, the reduced levels in D-lactate and DAO were observed in the AOS500 group, indicating that AOS500 inclusion preserved the integrity of the ileal epithelial barrier not only by elevating TJs expression but also by reducing intestinal permeability. Similarly, piglets fed AOSs also showed lower intestinal permeability, suggesting that AOSs improved intestinal barrier function and prevented invasions by pernicious luminal microbes and bacterial metabolites [17].

The mucus barrier is the first-line defensive layer that separates environmental and infectious agents from epithelial cells [38]. It is comprised of mucus produced by goblet cells and overlies intestinal epithelial cells. The mucus barrier in the small intestine is a single layer that acts as a diffusion barrier for antimicrobial proteins and peptides and enables nutritional uptake [39]. The large intestine is comprised of two distinct layers: an inner layer, which acts as a barrier against luminal bacteria, and an outer layer, which permits bacterial colonization [40]. It has been shown that goblet cells and mucin play a critical role in intestinal barrier function. The importance of mucin in piglets was demonstrated by experiments in a diarrheal pig model, which found that diarrhea led to a compromised intestinal barrier by reducing counts of goblet cells and mucin expression [41]. Our results showed that the number of goblet cells was obviously improved by supplementations with 250 mg/kg AOS compared with the CON group, indicating a potential role for AOSs in enhancing the mucus barrier, thereby reinforcing the intestinal barrier function, which is in line with a previous study showing that the enumeration of goblet cells was elevated by AOSs [42], galacto-oligosaccharides [43], and polysaccharides [44]. These results show that administering AOSs is an effective way to modulate the intestinal mucus barrier, similar to other oligosaccharides and polysaccharides, indicating that AOSs play critical role in the development of the pig breeding industry.

The impairment of mucus layer often results in the exposure of gut microbiota and harmful substances to immune cells, ultimately leading to intestinal inflammation [39]. It is well established that early weaning is related to an intestinal inflammatory response in piglets. Weaned piglets have been reported to be prone to intestinal inflammation, which involved an elevation in pro-inflammatory cytokines and a reduction in anti-inflammatory cytokines [27,41]. The intestinal development of infants was shown to be susceptible to a high concentration of TNF-α, as evidenced by elevated numbers of TNF-α^+^CD4^+^ T cells and the expression of TNF-induced genes in the afflicted intestinal tissues [45]. The excessive production of IL-1β has been shown to be involved in the development of inflammation and colitis [46,47]. We found that dietary supplementation with 500 mg/kg AOS significantly decreased the levels of TNF-α in the serum and ileum as well as ileal IL-1β, which suggested suppressed inflammatory reactions in piglets. Our work is in line with earlier reports, in which weaned piglets supplemented with AOSs improved fumonisin B1-induced systemic inflammation by decreasing the levels of pro-inflammatory cytokines IL-1β, TNF-α, and IFN-γ [48]. Furthermore, the inflammatory microenvironment can impair the epithelial barrier by destroying the structure and function of epithelial intercellular junctions, leading to intestinal barrier dysfunction and mucosal function dysbiosis [49]. Notably, the number of goblet cells and the activity of DAO was correlated with levels of IL-10 and IL-6 in our study, providing further support that AOSs could protect intestinal barrier integrity by alleviating the inflammatory responses to maintain intestinal mucosal barrier function. However, further studies in piglets will be needed to clarify these findings and to explore the mechanism of the modulation of intestinal barrier function by AOSs. Accordingly, several studies on piglets have also shown correlations between cytokines IL-1β and barrier function-related markers [50,51], highlighting the connection with the inflammatory microenvironment, intestinal barrier function, and mucosal homeostasis.

Normally, the antioxidant defense systems and oxidation defense system are in dynamic equilibrium [52]. Weaning has been found to induce severe oxidative stress in piglets, as it disrupts the equilibrium between oxidants and antioxidants. This disruption further hampers the development of intestinal barrier function [1,53]. The antioxidant enzymes, including T-SOD, CAT, and GSH-Px, play a pivotal role in counteracting oxidative damage. T-AOC indicates the overall antioxidant ability of the defense system. T-SOD and GSH-Px help the host to prevent peroxide damage by reducing antioxidants [54,55]. CAT is the key antioxidant enzyme that decomposes hydrogen peroxide into oxygen and water [56]. As an end product of lipid peroxidation, MDA is a biomarker of cellular oxidative stress [57]. In our study, the elevated levels of T-AOC, T-SOD, and CAT as well as reduced MDA levels in the serum of AOS500 supplemented-piglets were observed, indicating that using 500 mg/kg AOS improved the antioxidant capacity of weaned piglets. Similar to our findings, several studies have demonstrated that AOSs and other functional oligosaccharides can act as a prebiotic to elevate levels of antioxidant enzymes, thus enhancing antioxidant capacity [21,58]. Furthermore, improved intestinal barrier function has been associated with antioxidant capacity. Tao et al. [59] pointed out that chitosan oligosaccharides could alleviate lipopolysaccharide-induced oxidative stress by lowering the degree of lipid peroxidation and restoring the activities of antioxidant enzymes, thereby maintaining the intestinal barrier function. A dietary combination of multiple saccharides (xylo-oligosaccharides and gamma-irradiated astragalus polysaccharides) could provide broilers with a better redox environment and more complete intestinal mucosal barrier function [60]. According to these results, AOS inclusion improved the function of the intestinal barrier function in weaned piglets, possibly by enhancing antioxidant capacity.

The host–microbiota crosstalk is integral for maintaining and regulating intestinal homeostasis for both the host and microbiota, and also plays an important role in the systemic health of the host [61]. The microbiome is maintained and shaped by many factors including daily diet fluctuations [62]. Consequently, the alteration of the structural composition and metabolism of the gut microbiota induced by AOSs was investigated in our study. Specific bacteria (e.g., Helicobacter, Catenisphaera, and Eubacterium_siraeum_group) were identified to be influenced by AOSs in the colon. *Helicobacter*, the relative abundance of which was decreased in the AOS treatment group in the study, is thought to be a pathogenic bacterium and the increased abundance of Helicobacter is representative of gut dysbiosis [63]. Its infection is a strong causal promoter of colorectal carcinogenesis [64]. Certain species within the *Helicobacter* genera have the capability to degrade the oligomeric structure of mucins in the intestine. This, in turn, permits the penetration of enteropathogens through the protective mucus barrier, ultimately triggering intestinal inflammation [65]. The strong pro-inflammatory response induced by *Helicobacter* in the small intestine was accompanied by the activation of NF-κB and STAT3 pathways [64]. *Helicobacter* has also been shown to correlate with the production of intestinal reactive oxygen species [66]. In this study, Helicobacter was positively related with pro-inflammatory cytokines (TNF-α and IL-1β) and negatively correlated with antioxidant enzymes (T-AOC, T-SOD, and CAT), indicating that the ingestion of AOSs modulates the gut microbiota, inhibiting pathogens including Helicobacter, which in turn might affect the immune system and antioxidant capacity, thus keeping the gut ecosystem in balance. Furthermore, *Escherichia_Shigella* is the most commonly pathogenic bacteria that caused piglet diarrhea, thereby impairing the intestinal barrier function [41]. In our study, both AOS250 and AOS1000 treatment significantly reduced the relative abundance of the harmful bacteria *Escherichia_Shigella* in piglets, further supporting AOSs as protective agents against intestinal barrier dysfunction [67]. It has been demonstrated that *Faecalibacterium* has anti-inflammatory properties on compromised barrier function and improves the dysbiosis of the gut microbiota [68]. A previous study revealed that *Faecalibacterium* modulated by probiotics was negatively correlated with pro-inflammatory cytokines, exhibiting an anti-inflammatory effect [69]. These results were consistent with our findings, according to which *Faecalibacterium* may be a positive indicator of the immune response of the host, since it had a positive correlation with IL-10 and goblet cells. Xie et al. also observed an increase in the relative abundance of *Faecalibacterium* when dextran sulfate sodium-induced mice were given polysaccharides from tea; this was accompanied by reductions in inflammation and the repairing of the intestinal barrier function [44].

SCFAs are produced by microbial fermentation and can be rapidly absorbed by the host’s gut epithelium to provide the host with energy. SCFAs mainly consist of acetate, propionate, and butyrate, which account for more than 95%, and the rest of the ingredients are valerate, isobutyrate, and isovalerate [70]. Acetate, propionate, and butyrate are produced by specific gut microbiota via different metabolic pathways. Acetate production pathways are widely distributed in anaerobic bacteria [71]. Propionate is mainly synthesized by many enteric bacteria, such as *Bacteroidetes* spp., *Veillonella* spp., *Proteobacteria*, and *Ruminococcus obeum* via the succinate pathway, the acrylate pathway, or the propanediol pathway. Our results suggested a potential role of *Veillonella* induced by AOSs (at the level of 500 mg/kg and 1000 mg/kg) in driving the elevated levels of propionate, thus supplying energy for the host and favoring gut microenvironment homeostasis. Butyrate is produced by some bacteria such as *Faecalibacterium prausnitzii*, *Eubacterium* spp., *Roseburia* spp. and by phosphotransbutyrylase and butyrate kinase or the butyryl-CoA–acetate CoA-transferase route [70,72]. Among the SCFAs, butyrate is the most preferred source of energy for the host, and is produced by the bacterial fermentation of carbohydrates in the intestine [73]. The insignificant difference in butyrate production in our study could have been due to the low dietary fiber as the main substrate, masking responses among the AOS treatments. Additionally, the content of butyrate in the CON and the AOS group did not differ significantly, which suggests that butyrate and its energy substrates are more likely to be absorbed and utilized by the host [74].

## 4. Materials and Methods

### 4.1. Study Animals and Experimental Design

A total of 144 weaned piglets (Duroc × Landrace × Large White, with an average body weight of 7.79 ± 0.01 kg) were randomly assigned into 4 dietary treatments according to body weight and sex; treatments included a control diet (CON), and 3 treated diets formulated with 250 mg/kg, 500 mg/kg, and 1000 mg/kg AOS, respectively. The AOSs were purchased from Qingdao BZ Oligo Biotech Co., Ltd. (purity > 95%, Shandong, China). AOSs consist of ꞵ-D-mannuronic acid (M) and α-L-guluronic acid (G). The molecular weight, M/G ratio, and degree of polymerization were 2.21 kDa, 1.6, and 2–5 [75]. In each dietary treatment, 6 replicates and 6 piglets per replicate were used. The ingredient composition and nutritional levels of the diets are shown in Table S1. The diets were formulated according to the recommendations of Nutrient Requirements of Swine. Water was provided ad libitum to the piglets. The experimental period was 28 days. At d 14 and d 28, the feed consumption and body weight (BW) were recorded to determine ADFI, ADG, and G:F ratio.

### 4.2. Sample Collection and Processing

At d 28 of the experiment, blood samples were collected from 6 piglets from each group via jugular vein puncture and centrifuged to separate serum. The serum was kept at −80 °C. Then, the piglets were euthanized using electrical stunning. After exsanguination, the ileum was collected. A total of 0.5–1 cm of the middle section was fixed in 4% paraformaldehyde solution or 2.5% glutaraldehyde. After gently flushing the ileum with saline three times, the mucosa was collected and frozen immediately in liquid nitrogen. Mucosal samples were stored at −80 °C for future analysis.

### 4.3. Chemical Analysis

The gross energy (GE) was measured using an adiabatic bomb calorimeter according to the method of ISO 9831:1998 [76]. The crude protein was analyzed using method GB/T6432 [77]. Amino acids were determined using an amino acid analyzer (L8800, Hitachi, Tokyo, Japan). Calcium and phosphorus were detected using an inductively coupled plasma emission spectrometry instrument (Avio 200, PerkinElmer, Waltham, MA, USA).

### 4.4. RNA Isolation and Quantitative Real-Time PCR (qRT-PCR)

Total RNA from the ileal mucosa was extracted using a commercial kit (R013-50, GeneBetter, Beijing, China) according to the instructions. RNA reverse transcription and quantitative real-time PCR were performed using the commercial kits (PrimeScriptTMRT reagent kit with gDNA eraser, SYBR Premix Ex Taq II kit, Takara-Bio, Dalian, China). The relative expression of genes was measured using a CFX96 real-time system (Bio-Rad, Hercules, CA). The target gene expression was calculated using the 2^−ΔΔCT^ method, and the value was normalized to the housekeeping gene β-actin. Table S2 shows the primer sequences for samples.

### 4.5. Intestinal Histological Evaluation

Fixed tissues in 2.5% glutaraldehyde were washed with PBS 3 times, 15 min each. Then, the tissues were transferred into 1% osmium tetroxide for 2 h at room temperature. The washing process with PBS was repeated 3 times, 15 min each. Following ethanol dehydration with gradient, storage in ter-Butyl alcohol, installation of quick-drying silver paint and gold-palladium coating, the tissues underwent examination using SEM.

Fixed tissues in 4% paraformaldehyde solution were embedded in paraffin and 5 μm sections were counterstained with hematoxylin and eosin (HE). For histological analysis, the sections were carried out with a light microscope and Image J software (https://imagej.net/ij/, accessed on 1 July 2024). The sections were stained with AB-PAS following the manufacturer’s instructions for goblet cells quantitatively. The slides were visualized with a DM300 light microscope. The number of goblet cells were counted manually.

### 4.6. Biochemical and Immunological Parameters in Serum

LPS in the serum was measured using a commercial assay kit (Xiamen Bioendo Technology Co., Xiamen, China). The activity of DAO and the level of D-lactate were measured in accordance with the manufacturing instructions (Nanjing Jiancheng Bioengineering Institute, Nanjing, China). T-SOD, MDA, CAT, T-AOC, and GSH-Px in serum were detected according to the instructions of the kits. The levels of IL-6 and TNF-α were measured using the ELISA kit following the manufacturer’s protocol (Cusabio, Wuhan, China).

### 4.7. 16S rRNA Gene Sequencing and Analysis

16S rRNA V3-V4 variable regions were amplified using PCR using primers 338F and 806R and then sequenced using the Illumina MiSeq sequencing platform. The aforementioned analysis was performed using the online platform (Majorbio I-Sanger Cloud Platform). To evaluate taxa diversity and evenness, Shannon’s index, Chao1, Simpson, and ACE were calculated. The PCoA and DESeq2 analysis was processed using the web-based tool MicrobiomeAnalyst.

### 4.8. Quantitative Analysis of SCFAs

Gas chromatography (GC) was used to quantify SCFAs in the colonic luminal contents [78]. Briefly, the chyme of the colon was mixed with ultrapure water and then centrifuged. The supernatant was filtered through a filter membrane, metaphosphoric acid was added (25%, *w*/*v*), and it was then centrifuged and filtered once again. Finally, it was subjected to the GC system.

### 4.9. Statistical Analysis

Statistical analysis was performed using sing JMP 13.0 (SAS Institute, Inc., Cary, NC, USA). Statistical differences were determined using the one-way ANOVA with the Tukey HSD test. Results were presented as the means with standard error of the mean. The *p* values below 0.05 were considered statistically significant. *p* values between 0.05 and 0.10 were used to indicate a tendency. PLS-DA and Data Integration Analysis for Biomarker discovery using Latent variable approaches for Omics studies (DIABLO) analysis was carried out using the package “mixOmics” in R program (Version 4.3.1).

## 5. Conclusions

In conclusion, we observed that dietary AOS supplementation plays a beneficial role in the intestinal health of piglets. The 500 mg/kg of AOS supplementation exhibited growth-promoting effects as indicated by the increased final BW and ADG of piglets. The AOS treatment reinforced the intestinal barrier function by increasing the ileal villus height, density, and fold as well as the expression of tight junction proteins, especially at the dose of 500 mg/kg AOS. The potential dose of AOSs for weaned piglets was established as 500 mg/kg for promoting beneficial gut health. AOS500 supplementation further improved intestinal permeability by reducing the level of DAO and D-lactate. Meanwhile, supplementations with AOSs showed positive effects on enhancing antioxidant capacity and alleviating intestinal inflammation by elevating the levels of antioxidant enzymes and inhibiting excessive inflammatory cytokines. In addition, the DESeq2 analysis showed that AOS supplementation inhibited the growth of harmful bacteria Helicobacter and *Escherichia_Shigella*, and enhanced the relative abundance of *Faecalibacterium* and *Veillonella*. These two bacteria were further found to be correlated with the reduced level of pro-inflammatory cytokines and higher production of propionate, which explains the modulating effects of AOSs on the gut microbiota and their metabolite production.

## Figures and Tables

**Table 1:** Effects of AOS on the growth performance of piglets.

	Diet				*p* Values		
Item	CON	AOS250	AOS500	AOS1000	Diet	Linear	Quadratic
Initial BW, kg	7.78 ± 0.02	7.77 ± 0.02	7.80 ± 0.01	7.81 ± 0.02	0.453	0.771	0.120
Day 14 BW, kg	11.58 ± 0.15	12.12 ± 0.22	12.82 ± 0.48	12.04 ± 0.39	0.106	0.128	0.099
Final BW, kg	19.18 ^b^ ± 0.48	20.05 ^ab^ ± 0.36	22.35 ^a^ ± 1.06	20.29 ^ab^ ± 0.77	0.036	0.183	0.028
Day 1–14 ADG, kg/day	0.27 ± 0.01	0.31 ± 0.01	0.36 ± 0.03	0.30 ± 0.03	0.118	0.127	0.120
Day 15–28 ADG, kg/day	0.54 ^b^ ± 0.02	0.57 ^ab^ ± 0.03	0.68 ^a^ ± 0.04	0.59 ^ab^ ± 0.04	0.046	0.292	0.027
Day 1–28 ADG, kg/day	0.41 ^b^ ± 0.02	0.44 ^ab^ ± 0.01	0.52 ^a^ ± 0.04	0.45 ^ab^ ± 0.03	0.039	0.164	0.032
Day 1–14 G:F	1.56 ± 0.09	1.47 ± 0.05	1.53 ± 0.09	1.40 ± 0.07	0.523	0.674	0.558
Day 15–28 G:F	1.39 ± 0.14	1.50 ± 0.08	1.36 ± 0.04	1.49 ± 0.05	0.570	0.606	0.774
Day 1–28 G:F	1.45 ± 0.10	1.48 ± 0.06	1.42 ± 0.04	1.46 ± 0.04	0.906	0.796	0.665
Day 1–14 ADFI, kg	0.42 ± 0.03	0.45 ± 0.02	0.54 ± 0.05	0.42 ± 0.04	0.096	0.202	0.261
Day 15–28 ADFI, kg	0.75 ± 0.07	0.85 ± 0.06	0.92 ± 0.05	0.87 ± 0.04	0.224	0.175	0.114
Day 1–28 ADFI, kg	0.59 ± 0.05	0.65 ± 0.04	0.73 ± 0.04	0.64 ± 0.03	0.114	0.123	0.102

AOS: alginate oligosaccharide, BW: body weight, ADG: average daily gain, G:F: gain: feed, ADFI: average daily feed intake. Data are presented as means ± SEM. Different letters between the groups are statistically significant (*p* < 0.05).

## Data Availability

The sequences generated in this study are available in the NCBI Sequence Read Archive database (Accession Number: PRJNA989757). The datasets used in this manuscript are available from the corresponding author on reasonable request.

## Supplementary Materials

The following supporting information can be downloaded at: https://www.mdpi.com/article/10.3390/ijms25158029/s1.
