# Supplementary material for: Alginate Oligosaccharides Enhance Antioxidant Status and Intestinal Health by Modulating the Gut Microbiota in Weaned Piglets
Source: Int J Mol Sci. 2024 Jul 23;25(15):8029. doi: 10.3390/ijms25158029 (PMC11311613; doi:10.3390/ijms25158029)
Supplement: Supplementary file 1 [file ijms-25-08029-s001.zip › ijms-3085052-supplementary.pdf]

International Journal of *Molecular Sciences*

MDPI

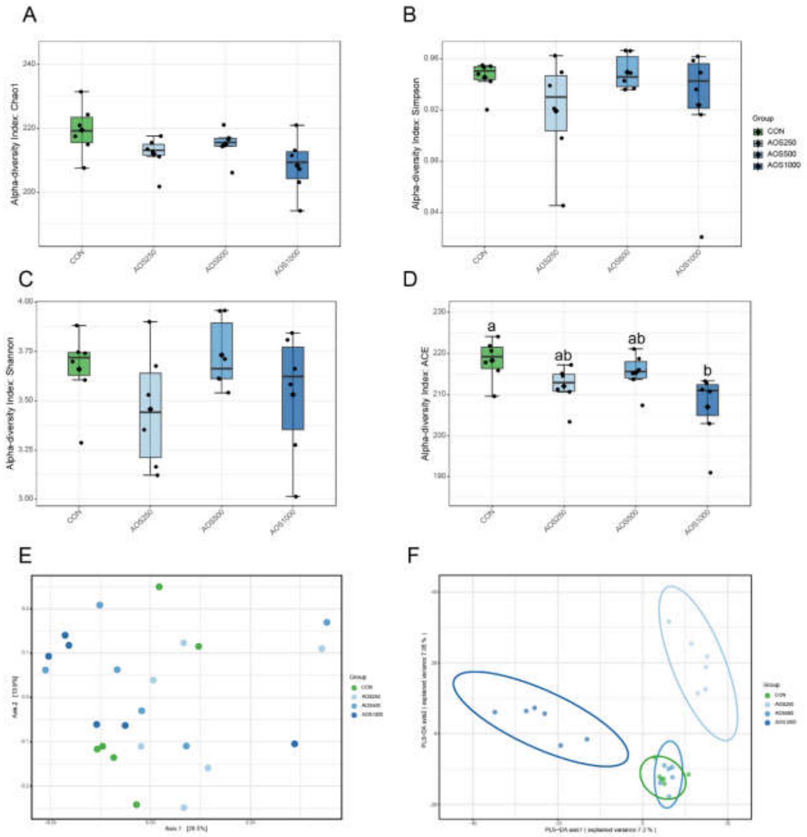

Figure S1. The gut microbial composition of piglets. Plots of α-diversity as measured by Chao1 (A), Simpon (B), Shannon (C), and ACE (D) at genus level. PCoA (E) and PLS-DA (F) analysis of the colonic microbiome at genus level.

Table S1. Composition and nutrient levels of the basal diet (air-dry basis).

Items	Content	Items	Content
**Ingredients, %**		**Nutrient levels**	
Corn	38.00	DE, kcal/kg	3459
Extruded soybean	18.50	CP, %	19.62
Soybean meal (CP%)	13.00	Ca, %	0.93
Fish meal	5.00	Total P, %	0.66
Whey powder	12.00	Lys, %	1.39
Soybean oil	0.72	Met, %	0.49
Calcium phosphate	0.78	Thr, %	0.87
Limestone	1.00	Trp, %	0.22
Sodium chloride	0.30		
DL-Methionine	0.16		
L-lysine	0.32		
L-Threonine	0.11		
L-Tryptophan	0.01		
Premix[1]	0.10		

1 Vitamin and mineral premixes were supplied per kg of diet as-fed: vitamin A , 3300 IU; vitamin D, 3300 IU; vitamin E, 22.5 mg; vitamin K3, 3 mg; vitamin B1, 30 mg; vitamin B2, 12 mg; vitamin B6, 4.86 mg; vitamin B12, 0.03 mg, nicotinic acid, 30 mg; calcium pantothenic acid, 33.12 mg; folic acid, 1.746 mg; biotin, 0.48 mg; Fe (FeSO4), 140.0 mg; Cu (CuSO4·5H2O), 20.0 mg; Zn (ZnSO4), 140.0 mg; Mn (MnSO4·H2O), 40 mg; I (KI), 0.5 mg; Se (Na2SeO3), 0.3 mg. DE: digestible energy; CP: crude protein; Lys: lysine; Met: methionine; Thr: threonine; Try: tryptophan; Ca: calcium; P: phosphorus.

Table S2. Primer sequences used for qRT-PCR.

Gene	Accession number	Primer sequences (5'-3')
β-actin	XM_003357928.4	F: GCGTAGCATTTGCTGCATGA
		R: GCGTGTGTGTAACTAGGGGT
ZO-1	XM_005659811.1	F: CTCCAGGCCCTTACCTTTCG
		R: GGGGTAGGGGTCCTTCCTAT
Occludin	NM_001163647.2	F: CAGGTGCACCCTCCAGATTG
		R: TATGTCGTTGCTGGGTGCAT
TNF-α	NM_214022.1	F: TAAGGGCTGCCTTGGTTCAG
		R: AGAGGTTCAGCGATGTAGCG
IL-1β	NM_214022.1	F: ATTCAGGGACCCTACCCTCTC
		R: CTTCTCCACTGCCACGATGA
IL-10	NM_214041.1	F: TCGGCCCAGTGAAGAGTTTC
		R: GGAGTTCACGTGCTCCTTGA
IL-6	NM_214399.1	F: CCCTGAGGCAAAAGGGAAAGAA
		R: CTCAGGTGCCCCAGCTACAT
